# Type 1 *CALR* mutation allele frequency correlates with CD34/CXCR4 expression in myelofibrosis-type megakaryocyte dysplasia: A mechanism of disease progression?

**DOI:** 10.1038/s41408-024-00991-2

**Published:** 2024-01-23

**Authors:** Giovanni Barosi, Rita Campanelli, Paolo Catarsi, Carlotta Abbà, Adriana Carolei, Margherita Massa, Robert Peter Gale, Vittorio Rosti

**Affiliations:** 1 Center for the Study of Myelofibrosis, Istituto di Ricovero e Cura a Carattere Scientifico Policlinico S. Matteo Foundation, Pavia, Italy; 2 General Medicine 2, Center for Sistemic Amyloidosis and High Complexity Diseases, Istituto di Ricovero e Cura a Carattere Scientifico Policlinico San Matteo Foundation, Pavia, Italy; 3 Centre for Haematology Research, Imperial College London, London, UK

**Keywords:** Haematopoietic stem cells, Oncogenesis

*Calreticulin* (*CALR*) mutations drive myeloproliferative neoplasms (MPNs). A 52-bp deletion (type 1) and a 5-bp insertion (type 2) are the most common mutations, but others can also be classified into type 1-like and type 2-like mutations. *CALR* mutation sub-types contribute to disparate MPN phenotypes and prognoses [1–3]. The distinct molecular mechanisms and clinical differences between type 1 and type 2 *CALR* mutations are elusive [4, 5].

Recently, it was reported that expression of S100A8, a calcium-binding protein implicated in proliferation, differentiation, and apoptosis of several cell types, is increased in type 1 *CALR*-mutated MPN-model cells [6]. It is suggested the key to the divergent S100A8 concentrations results from an epigenetic alteration, due to the different methylation status of *CALR* harboring type 1 mutation.

We hypothesized that *CALR* type 1 mutation could epigenetically de-regulate the expression of proteins involved in the progression of MPNs other than S100A8. To test this hypothesis, we analyzed the influence of *CALR* mutation sub-types on the chemokine receptor CXCR4, a key regulator of homing, retention, and quiescence of hematopoietic stem cells (HSCs). The choice of CXCR4 was determined by data indicating that in myelofibrosis (MF) *CXCR4* transcription is regulated by abnormal methylation of the *CXCR4* promoter [7]. Reduced CXCR4 surface expression on CD34-positive hematopoietic stem and progenitor cells (CD34/CXCR4) is associated with a briefer interval to disease progression, blast transformation, and death [8].

We interrogated a cohort of 188 subjects with *CALR* mutation classified as myelofibrosis-type megakaryocyte dysplasia (MTMD), including 48 with clonal megakaryocyte dysplasia with isolated thrombocytosis (CMD-IT—falling into the category of MPN-unclassifiable according to the WHO classification [9]), 54 with prefibrotic myelofibrosis (pre-MF) and 86 with overt myelofibrosis (overt-MF). Diagnoses of pre-MF and overt-MF were based on operative WHO diagnostic criteria at the time of their first visit and re-classified according to 2022 revised criteria [10]. CMD-IT subjects were otherwise classified using adjudicated criteria [11].

We derived CD34/CXCR4 expression values and healthcare data by reviewing the results of subjects referred to the Center for the Study of Myelofibrosis at the IRCCS Policlinico S. Matteo Foundation in Pavia. The inclusion of subjects in the institutional database had been approved by the IRCCS Policlinico S. Matteo Foundation’s Institutional Ethics Committee and subjects had given their written informed consent (Reference 20110004143 of the 26.9.2011).

*CALR* mutation was assayed by PCR amplification and capillary gel electrophoresis starting from granulocyte DNA. *CALR* mutation variant allele frequency (VAF) was determined by automated interpolation of the area under the curve and expressed as the ratio between the mutant peak area and the sum of mutant and wild-type peak areas × 100. CXCR4 expression analyses were done on blood collected in EDTA tubes and incubated with fluorochrome-labeled antibodies as described [12].

Characteristics of the *CALR*-mutated subjects at the time of diagnosis are summarized in Table 1. Type 1 and type 1-like (called type 1) and type 2 and type 2-like (called type 2) *CALR* mutations were detected in 129 (71%) and 53 (29%) of the cohort. Subjects with overt MF or pre-fibrotic MF had a higher proportion of type 1 compared with type 2 mutations [77% vs. 23% (*P* < 0.001) and 79% vs. 21% (*P* < 0.001)]. In contrast, subjects with CMD-IT had a higher proportion of type 2 *CALR* mutations compared with type 1 [53% vs. 47% (*P* = 0.53)].

**Table 1:** Clinical and laboratory characteristics of myelofibrosis-type megakaryocyte dysplasia (MTMD) subjects stratified according to the *CALR* mutation subtypes.

	Total	Type 1 mutation	Type 2 mutation	Comparison between type 1 and type 2 mutations
*n* (%)	182	129 (70.9)	53 (29.1)	
Age, years, median (IQR)	44 (35–57)	44 (35–54)	45 (36–57)	*P* = 0.31
Male, *n* (%)	112 (61.5)	84 (65.1)	28 (52.8)	OR = 1.67; 95% CI, 0.87–3.19; *P* = 0.12
Hb, g/dL, median (IQR)	12.9 (11.1–13.8)	12.8 (11–13.7)	13.2 (11.3–14)	*P* = 0.16
WBC x 10E + 9/L, median (IQR)	7.9 (6.4–9.7)	7.7 (6.3–9.9)	8 (6.7–9.6)	*P* = 0.83
Monocyte count × 10E + 9/L, median (IQR)	504 (348–678)	500 (351–657)	506 (342–650)	*P* = 0.99
Platelet × 10E + 9/L, median (IQR)	652 (430–885)	636 (352–831)	717 (529–959)	*P* = 0.04
Spleen size, cmE+2, median (IQR)	100 (90–140)	110 (90–165)	90 (90–123)	*P* = 0.02
LDH, ×ULN, median (IQR)	1.56 (1.01–2.26)	1.67 (1.19–2.33)	1.08 (0.87–1.83)	*P* = 0.01
CD34 × 10E + 6/L, median (IQR)	*n* = 81 23.9 (6.3–60)	*n* = 58 22.8 (6.3–60)	*n* = 23 28.5 (5.8–60.3)	*P* = 0.94

Data refers to the diagnosis of the disease, i.e. at the time of the diagnostic bone marrow biopsy.

*IQR* Interquartile range, *LDH* lactic dehydrogenase, *UPN* upper limit of normal.

At diagnosis, subjects with type 1 CALR mutation had less severe thrombocytosis compared with those with type 2 mutations (median, 636 × 10E + 9/L vs. 717 × 10E + 9/L; *P* = 0.04). Also, spleen size was larger and lactate dehydrogenase (LDH) concentration higher in subjects with type 1 mutation (*P* = 0.02 and *P* = 0.01).

At Cox regression analysis, *CALR* mutation type was not associated with significant differences in the incidence of anemia, leukocytosis, splenomegaly, blast transformation, and death. However, WBC concentration <4 × 10E + 9/L and platelet concentration <150 × 10E + 9/L occurred earlier in subjects with type 1 *CALR* mutation [median time to event, 288 months vs. 378 months; hazard ratio (HR), 2.19; 95% confidence interval (CI), 0.74–6.48; *P* = 0.14, and 297 months vs. not reached; HR, 1.72; 95% CI, 0.77–3.80; *P* = 0.16, respectively].

In the cross-sectional collection of data referring to subjects analyzed at diagnosis and during follow-up (*n* = 170), type 1 *CALR* mutants (*n* = 119) had a higher VAF compared with those with a type 2 mutation [*n* = 51; medians, 46%; Interquartile Range (IQR), 41–52% vs. 44%; IQR, 39–47%; *P* = 0.004; means, 49%; standard deviation (SD), 16.7% vs. 41%; SD, 9.6%; *P* = 0.001). Increasing *CALR* mutation VAF was correlated with co-variates associated with disease progression such as decreased hemoglobin (*r* = −0.42; *P* = <0.001) and platelet concentrations (*r* = −0.32; *P* = 0.001), increased spleen size (*r* = 0.36; *P* < 0.001), WBC (*r* = 0.27; *P* = 0.006), blood monocytes (*r* = 0.33; *P* = 0.002), and blood CD34-positive cells concentrations (*r* = 0.32; *P* = 0.002). In subjects with type 1 *CALR* mutation, these correlations in the unselected population persisted with a Pearson correlation coefficient of VAF with these parameters ranging from 0.27 to 0.43, with the highest values for hemoglobin and platelet concentration. In contrast, in subjects with a type 2 mutation, associations between increasing VAF and lower hemoglobin concentration remained (*r* = −0.37; *P* = 0.040), but correlations with WBC, platelet, and monocyte concentrations, spleen size, and proportion of CD34-positive blood cells were no longer significant (Pearson correlation coefficients of −0.001 to 0.24, *P* values from 0.19 to 0.96).

In the cross-sectional collection of samples (*n* = 161) median blood CD34/CXCR4 expression was 50% (IQR, 33–74%). CD34/CXCR4 expression clustered differently in type 1 (*n* = 112; median, 44%, IQR, 30–61%) compared with type 2 *CALR* mutation (*n* = 49; median, 74%; IQR, 51–89%; *P* < 0.001). CD34/CXCR4 expression was inversely correlated with *CALR* mutation VAF (*r* = −0.36; *P* < 0.001). However, this correlation was only for the type 1 mutation (*r* = −0.41; *P* < 0.001), not type 2 (*r* = 0.00; *P* = 0.99; Fig. 1).

**Fig. 1:**
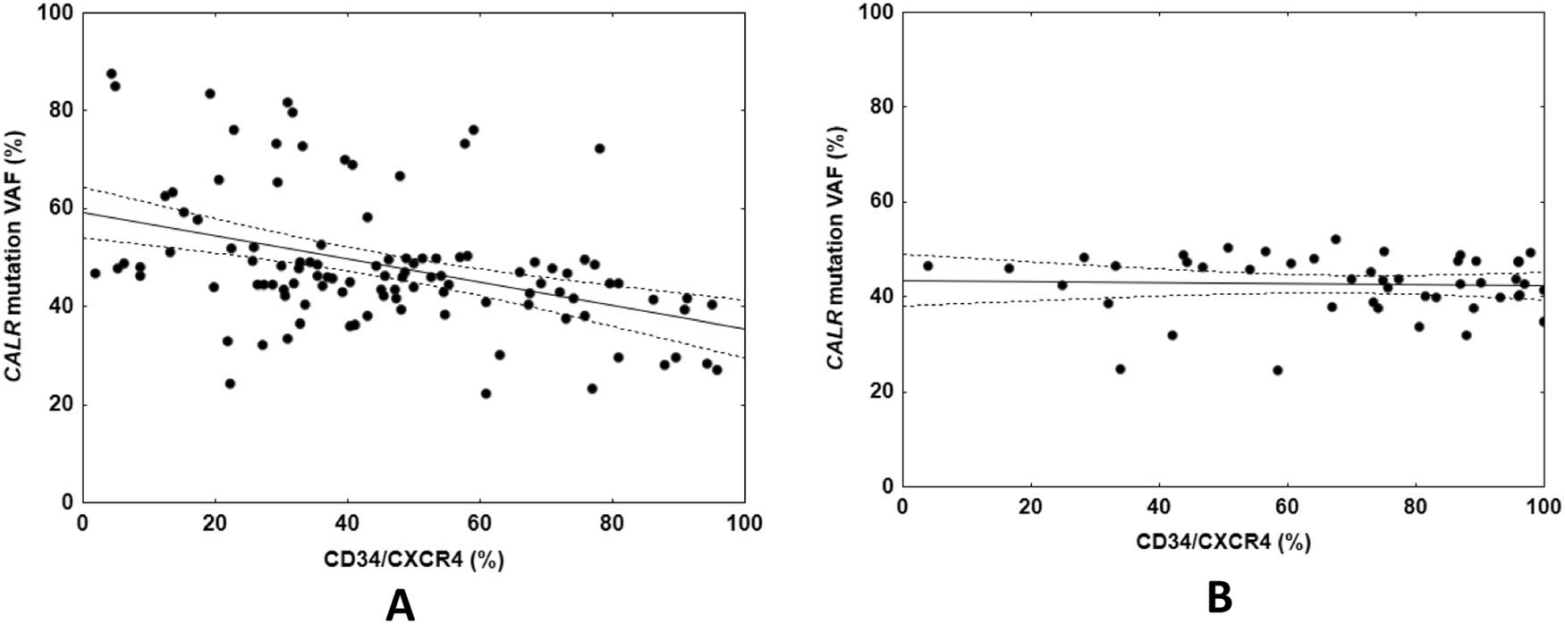
Correlations between CALR mutation variant allele frequency (VAF) and CXCR4 expression on CD34+ cells (CD34/CXCR4). Panel **A**: in subjects with CALR type 1 mutation; Panel **B**: In subjects with CALR type 2 mutation.

Taking account of co-variates implicated in the relation between blood CD34/CXCR4 expression and *CALR* mutation VAF, CD34/CXCR4 expression resulted lower in males (*n* = 94) compared with females (*n* = 67; 45%, IQR, 31–61% vs. 67%, IQR, 37–83%; <0.001) and was significantly correlated with age (*r* = −0.18; *P* = 0.02). In a logistic multi-variable regression analysis, CD34/CXCR4 expression <39% (the lower reference interval limit of CD34/CXCR4 expression in normals [8]) correlated with *CALR* mutation type (adjusted odds ratio [OR] = 0.23 (0.07, 0.72; *P* = 0.01).

In summary, we found type 1 *CALR* mutation is more common in the more severe MTMD category, i.e. overt MF, is associated with lower platelet and higher LDH concentrations, larger spleen size, and earlier development of leukopenia and thrombocytopenia compared with type 2 mutations. Some but not all of these correlations are reported by others [13–15]. We also found a strong correlation between type 1 *CALR* mutation VAF and indicators of disease progression, especially anemia and thrombocytopenia. These data reinforce the impact of type 1 *CALR* mutation on disease phenotype and trajectory, even though we did not find any influence on survival or progression to leukemia.

In our hypothesis-driven analyses, we first provide evidence of a dependence of CD34/CXCR4 expression on the *CALR* mutation type. Our study is retrospective and observational, so we can only infer causality between type 1 *CALR* gene expression and reduced CXCR4 expression. However, the association of type 1*CALR* mutation and low CD34/CXCR4 expression remained after adjusting for other co-variates associated with decreased CXCR4 regulation. Furthermore, type 1 *CALR* mutation VAF and down-regulated CD34/CXCR4 expression respected a dose–response relationship. These characteristics of the results give support to the causality.

This study suffers from limitations. Not all cases had all parameters useful for the analysis. However, our study is of a consecutive series of cases from an institutional database that systematically collects the most important disease co-variates.

Our study provides an innovative perspective on how CD34/CXCR4 may be differentially regulated in type1 *CALR*-mutated MTMD, a finding with potential implications for predicting prognosis and for therapy. Comparative studies through targeted and methylation sequencing can further clarify the epigenetic diversity between type 1 and 2 *CALR*-mutated people with MTMD.

## Data Availability

Available on reasonable request from the corresponding author.

## References

[1] Tefferi A, Lasho TL, Finke C, Belachew AA, Wassie EA, Ketterling RP (2014). Type 1 vs type 2 calreticulin mutations in primary myelofibrosis: differences in phenotype and prognostic impact. Leukemia.

[2] Pietra D, Rumi E, Ferretti VV, Di Buduo CA, Milanesi C, Cavalloni C (2016). Differential clinical effects of different mutation subtypes in CALR-mutant myeloproliferative neoplasms. Leukemia.

[3] Kim HY, Han Y, Jang JH, Jung CW, Kim SH, Kim HJ (2022). Effects of CALR-mutant type and burden on the phenotype of myeloproliferative neoplasms. Diagnostics (Basel).

[4] Ibarra J, Elbanna YA, Kurylowicz K, Ciboddo M, Greenbaum HS, Arellano NS (2022). Type I but not type II calreticulin mutations activate the IRE1alpha/XBP1 pathway of the unfolded protein response to drive myeloproliferative neoplasms. Blood Cancer Discov.

[5] Arellano NS, Kurylowicz K, Maxwell L, Greenbaum HS, Ibarra J, Elf S (2021). Myeloproliferative neoplasm-associated type 2 calreticulin mutations differentially activate and depend on the ATF6 pathway of the UPR. Blood.

[6] Wang YH, Chen YJ, Lai YH, Wang MC, Chen YY, Wu YY (2023). Mutation-driven S100A8 overexpression confers aberrant phenotypes in type 1 *CALR*-mutated MPN. Int J Mol Sci.

[7] Bogani C, Ponziani V, Guglielmelli P, Desterke C, Rosti V, Bosi A, Myeloproliferative Disorders Research Consortium (2008). Hypermethylation of CXCR4 promoter in CD34+ cells from patients with primary myelofibrosis. Stem Cells.

[8] Barosi G, Rosti V, Catarsi P, Villani L, Abbà C, Carolei A (2021). Reduced CXCR4-expression on CD34-positive blood cells predicts outcomes of persons with primary myelofibrosis. Leukemia.

[9] Barosi G, Rosti V, Gale RP (2023). Myelofibrosis-type megakaryocyte dysplasia (MTMD) as a distinct category of BCR::ABL-negative myeloproliferative neoplasms. Challenges and perspectives. Leukemia.

[10] Khoury JD, Solary E, Abla O, Akkari Y, Alaggio R, Apperley JF (2022). The 5th edition of the World Health Organization Classification of Haematolymphoid Tumours: myeloid and histiocytic/dendritic neoplasms. Leukemia.

[11] Barosi G, Campanelli R, Massa M, Catarsi P, Carolei A, Abbà C (2023). Clonal megakaryocyte dysplasia with isolated thrombocytosis is a distinct myeloproliferative neoplasm phenotype. Acta Haematol.

[12] Rosti V, Massa M, Vannucchi AM, Bergamaschi G, Campanelli R, Pecci A (2007). The expression of CXCR4 is down-regulated on the CD34+ cells of patients with myelofibrosis with myeloid metaplasia. Blood Cells Mol Dis.

[13] Guglielmelli P, Maccari C, Sordi B, Balliu M, Atanasio A, Mannarelli C (2023). Phenotypic correlations of CALR mutation variant allele frequency in patients with myelofibrosis. Blood Cancer J.

[14] Benlabiod C, Cacemiro MDC, Nédélec A, Edmond V, Muller D, Rameau P (2020). Calreticulin del52 and ins5 knock-in mice recapitulate different myeloproliferative phenotypes observed in patients with MPN. Nat Commun.

[15] El-Khoury M, Cabagnols X, Mosca M, Vertenoeil G, Marzac C, Favale F (2020). Different impact of calreticulin mutations on human hematopoiesis in myeloproliferative neoplasms. Oncogene.

